# Genetic Polymorphism at 15 Codons of the Prion Protein Gene in 156 Goats from Romania

**DOI:** 10.3390/genes13081316

**Published:** 2022-07-23

**Authors:** Maria Rodica Gurau, Elena Negru, Teodor Ionescu, Anca Amalia Udriste, Călina Petruța Cornea, Stelian Baraitareanu

**Affiliations:** 1 Department of Clinical Sciences, Faculty of Veterinary Medicine, University of Agronomic Sciences and Veterinary Medicine of Bucharest, 105 Splaiul Independentei, District 5, 050097 Bucharest, Romania; mariagurau@fmvb.ro (M.R.G.); elena.negru@fmv.usamv.ro (E.N.); teodor.ionescu@fmv.usamv.ro (T.I.); 2 Laboratory of Molecular Plant Physiology, Research Center for Studies of Food Quality and Agricultural Products, University of Agronomic Sciences and Veterinary Medicine of Bucharest, 59 Marasti Blvd, District 1, 011464 Bucharest, Romania; amalia.udriste@qlab.usamv.ro; 3 Faculty of Biotechnologies, University of Agronomic Sciences and Veterinary Medicine of Bucharest, 59 Marasti Blvd, District 1, 011464 Bucharest, Romania; petruta.cornea@biotehnologii.usamv.ro

**Keywords:** genetic polymorphism, *PRNP*, scrapie, goat breeding programs

## Abstract

Background: The variability of prion protein gene (*PRNP*) codons and the frequency of alleles (K222, D146, and S146) that appear to confer genetic resistance to classical scrapie are still unknown in several goat populations/breeds prevalent in Romania. This work aims to assess the genetic polymorphism at 15 *PRNP* codons in Romanian goat populations to inform the development of goat breeding programs for scrapie resistance. Methods: Whole blood and hair follicles from Carpathian (50), French Alpine (53), and Banat’s White (53) breed goats were sampled to extract genomic DNA for genetic analyses and Sanger sequencing. In the targeted goat groups, one classical scrapie-positive Banat’s White goat was included. Results: The codons without polymorphisms were G37G, W102W, N146N, R151R, S173S, and I218I. The following non-synonymous polymorphisms of *PRNP* were recorded: P110P, P110S, P110T, T110T, G127G, G127S, I142I, I142M, T142I, H143H, P143P, R143R, R154R, H154R, P168P, Q168Q, Q211Q, Q211R, Q222Q, H222Q, K222K, S240S, P240P, P240S, and S240P. Conclusions: *PRNP* polymorphism was recorded in 60% (9/15) of codons. The scrapie-positive Banat’s White goat had G37G, W102W, T110T, G127G, I142I, H143H, N146N, R151R, R154R, P168P, S173S, R211R, I218I, Q222Q, and S240S. The K222 allele had a frequency of 6% (3/50) in Carpathian, 9.43% (5/53) in Banat’s White, and 15.09% (8/53) in French Alpine. Therefore, the polymorphisms detected in this sample of Romanian goat breeds are too rare to design a breeding program at the current time.

## 1. Introduction

The genotypes of the prion protein gene (*PRNP*) play an important role in the susceptibility of sheep and goats to scrapie, a naturally occurring transmissible spongiform encephalopathy (TSE) produced by prions [1]. While in sheep, numerous studies led to the development of national genetic selection plans in agreement with Regulation (EC) 999/2001 [2,3,4,5,6,7,8,9], there are insufficient studies involving local goat populations/breeds to validate Romanian control strategies. In the ovine selection programs for scrapie, the haplotype alanine/arginine/arginine (ARR) at codons 136, 154, and 171 is associated with decreased susceptibility or resistance to scrapie, whereas the haplotypes valine/arginine/glutamine (VRQ) and alanine/arginine/glutamine (ARQ) are linked with high susceptibility to scrapie [10].

The importance of active surveillance and control of TSEs has been generally accepted all over the world. European Union (EU) regulations set out general provisions for TSE prevention (monitoring systems, breeding programmes, prohibitions concerning animal feeding, specified risk material, and education programmes), control, and eradication measures (notification, measures with respect to suspect animals, measures following confirmation of the presence of a TSE, and contingency plans) [3]. The use of genetic screening programs in sheep increases the chances of eradicating scrapie in sheep. A similar approach should be applied to goats; however, this requires the accumulation of data concerning *PRNP* polymorphism in several local goat populations and breeds.

In the last 10 years, genetic variation of *PRNP* was reported in goat populations/breeds from Algeria [11], Canada [12], Ethiopia [13,14], Greece [15,16,17], Italy [18,19,20], Korea [21,22], Spain [23,24], Türkiye [25], Tunis [26], UK [27], and USA [28]. In addition to these reports, there is in-depth research on genetic resistance associated with some *PRNP* alleles, including those related to the K222 allele.

The highly protective effect of the K222 allele against classical scrapie has been demonstrated in several studies [29,30,31,32,33,34,35,36]. However, the European Food Safety Authority (EFSA) noted a very low frequency and heterogeneous distribution of the K222 allele in different goat breeds and EU Member States, which could lead to adverse effects on genetic diversity if a scrapie-resistance breeding programme were developed at the EU level [37]. Before developing a multinational resistance programme for scrapie in goats, each EU member state should assess the appropriateness of implementing national breeding programmes adapted to the genetic characteristics of the local goat population/breeds.

In addition, the *PRNP* polymorphisms at codons 21, 23, 37, 49, 102, 110, 127, 133, 137, 139, 142, 142, 143, 146, 146, 151, 154, 168, 173, 211, 218, 220, and 240 have been evaluated for scrapie genetic resistance in goats [15,16,17,22,26,30,31,32,33]. However, the EFSA summary results of goat genetic surveys in the EU emphasizes only codons 142, 146, 154, and 211–222 as codons with alleles of interest in breeding programmes to promote resistance to classical scrapie in goats. Insufficient data prevented a ranking of susceptibility at genotype level; however, the EFSA used a combination of the “weight of evidence” and the “strength of resistance” to rank the K222 allele with the highest resistance to classical scrapie, followed by D146, S146, Q211, H154, and M142 [37].

Considering the diseases produced by prions, the EFSA monitors the surveillance activities on TSE in cattle, sheep, goats, cervids, and other species by all Member States and some non-EU countries (the United Kingdom, Bosnia and Herzegovina, Iceland, Montenegro, North Macedonia, Norway, Serbia, and Switzerland). Every year, more than 300,000 sheep and 120,000 goats are tested in the EU [38,39,40]. In 2018, classical scrapie was confirmed in 1338 small ruminants (of which 38.69% were goats) and atypical scrapie in 119 small ruminants (of which 5.04% were goats) [38]. In 2019, classical scrapie was recorded in 1290 small ruminants (of which 29.38% were goats) and atypical scrapie in 97 small ruminants (of which 11.34% were goats) [39]. In 2020, there were 908 small ruminants (of which 35.13% were goats), while there were 107 small ruminants (of which 8.41% were goats) with atypical scrapie [40]. Up until 2017, 824 classical and zero atypical scrapie cases in sheep and goats were reported in Romania. Between 2018 and 2020, Romania was one of the most affected EU states by scrapie, with 206 cases in 2018 (out of 523 goats in the EU), 141 cases in 2019 (out of 390 goats in the EU), and 57 cases in 2020 (out of 328 goats in the EU) [38,39,40]. In Romania, only one atypical scrapie case was reported in 2020 (out of 26 goats with atypical scrapie in the EU) [40].

EU policy is designed to eradicate classical scrapie, which poses a higher risk of transmission between animals than atypical scrapie. The scientific report on atypical scrapie monitoring in 22 countries, published in 2021 by the EFSA, concluded that “there is no new evidence that atypical scrapie can be transmitted between animals under natural conditions, and it is considered more likely (subjective probability range 50–66%) that atypical scrapie is a non-contagious, rather than a contagious disease” [41].

This work aims to assess the genetic polymorphism at 15 codons (37, 102, 110, 127, 142, 143, 146, 151, 154, 168, 173, 211, 218, 222, and 240) of the *PRNP* in two local goat breeds (Carpathian and Banat’s White) and one cosmopolitan breed (French Alpine) reared in eastern Romania. Our research was justified by the lack of data on the susceptibility of Romanian goat populations to scrapie and whether the alleles K222, D146, and S146 that seem to confer genetic resistance to classical scrapie strains are present in Romanian goat populations. Furthermore, we aimed the amino acid polymorphism analysis at the same codons for a scrapie-positive Banat’s White goat.

## 2. Materials and Methods

### 2.1. Goat Herds, Breeds, and Biological Samples

#### 2.1.1. Goat Herds and Selection of Breeds

Goats from six farms located in eastern and south-eastern Romania were selected to meet the study’s objectives (Table 1). In Romania, goats are reared mainly in extensive low-input production systems that are oriented primarily towards milk production [42]. The 156 goats targeted in this study were from the main breeds grown in Romania: Carpathian (50), Banat’s White (53), and French Alpine (53). Goat ages were between 2 and 15 years.

The Romanian breed structure of goats is dominated by the indigenous breeds Carpathian and Banat’s White, which represent over 90% of the Romanian goats [42]. Carpathian goats seem to have a high degree of heterogeneity in meat production in relation to the rearing system [43] and the high quality of the meat in comparison to other breeds [44]. In addition, Carpathians have a remarkable organic resistance and adaptation to climatic conditions [42], milk yields between 220 and 350 kg/lactation, litter size between 130 and 160%, and growth rates in kids between 90 and 110 g/day [45,46]. These characteristics have led to the spread of the breed in Eastern European countries and to our interest in including it in this research. Banat’s White is currently listed as endangered and included in a genetic conservation program [42]. Banat’s White has a milk yield between 370 and 400 kg/lactation and a litter size between 200 and 225% [47]. French Alpine is one of the most popular exotic breeds in Romania, with a milk yield between 525 and 585 kg/lactation [48] and an average litter size of 115% [49].

#### 2.1.2. Samples Collection

Whole blood and hair follicles from each goat were sampled to extract genomic DNA using a protocol described by Gurău et al. (2021) [50]. Gurău et al. (2021) established that the hair follicles of goats can be used as a non-invasive method of sampling biological material for genomic DNA isolation (in whole blood and hair follicle samples, the DNA concentrations are enough for the specific amplification of 1200 bp DNA fragments). Whole blood was collected in a 5 mL sterile tube with K3-EDTA and the hair follicles in a 50 mL sterile transport tube with a screwcap (30–40 hair follicles/sample). The samples were transported in refrigerated conditions (+1–3 °C) within 24 h of sampling on the farm to the laboratory where they were stored at −20 °C until DNA extraction.

### 2.2. DNA Isolation and PRNP Gene Sequencing

#### 2.2.1. Genomic DNA Extraction

Genomic DNA extraction was conducted using PureLink Genomic DNA Kits (Invitrogen, Waltham, MA, USA) by using previously described protocols [50,51]. Briefly, 200 μL of whole blood collected on an EDTA sample and 30–40 hair follicles crushed with a sterile scalpel on a Petri dish were used for each animal to extract DNA in two different protocols described by the manufacturer.

#### 2.2.2. Total DNA Quantification

The amount of total DNA extracted was quantified with a fluorometer (Qubit 4 Fluorometer, Thermo Fisher Scientific Inc., Waltham, MA, USA) using Qubit dsDNA HS Assay Kits [52,53]. The protocol of quantification was previously described by Gurău et al. (2021) [50]. Briefly, the method measures the intensity of the signal from fluorescent dyes which are bound to the DNA molecule and the results are expressed in ng/µL.

#### 2.2.3. DNA Amplification of the *PRNP* Gene

Amplification was performed with Amplitaq Gold 360-DNA Polymerase-250U kits (Thermo Fisher Scientific, USA) in a 50-µL reaction volume, 25 mM MgCl2, 360 GC Enhancer, 40 μM dNTPs, 25 pmol/μL of each primer (Table 2), 10X AmpliTaqGold^®^ PCR Buffer, 2.5 units of AmpliTaq Gold^®^ (Applied Biosystems), and total DNA. The following thermal profile was used for sample amplification: 5 min at 96 °C, 30 s at 96 °C, 15 s at 57 °C, 90 s at 72 °C for 40 cycles, and a final cycle of 4 min at 72 °C. PCR products were analysed by 1.5% agarose gel electrophoresis (0.9 g agarose with 60 mL TAE/TBE 1× and 5 μL EtBr 10 mg/mL; 2 μL loading buffer with 10 μL amplicons; and migration at 90 V, 1.5 A, for 35 min). The fragment migrated at ~1200 bp.

#### 2.2.4. DNA Sanger Sequencing of the *PRNP* Gene

The sequencing reactions were performed by outsourcing to a specialized company (Antisel, Thessaloniki, Greece, CEMIA) using a Big Dye Terminator Cycle Sequencing Kit v3.1 (Thermo Fisher Scientific), ABI PRISM 3130 (Applied Biosystems), and primers previously described by Vaccari et al. (2009) and Migliore et al. (2015) (Table 3) [54,55].

### 2.3. Bioinformatics

Alignment and processing of the sequences were performed with BioEdit Sequence Alignment Editor, ver. 7.2.5 (Tom Hall, Ibis biosciences, Carlsbad, CA, USA). Sequences were aligned to the *Capra hircus* prion protein allelic variant (Accession number HM038415.1). All the electropherograms were checked at codons 37, 102, 110, 127, 142, 143, 146, 151, 154, 168, 173, 211, 218, 222, and 240 to identify mutations (Figure S1).

### 2.4. Statistical Analysis

Genotypic and allelic frequencies regarding codons 37, 102, 110, 127, 142, 143, 146, 151, 154, 168, 173, 211, 218, 222, and 240 were calculated for Carpathian, Banat’s White, and French Alpine breed goats. In this study, we were not focused on evaluating the possible inbreeding and transmission of unfavourable linked traits; the Hardy–Weinberg equilibrium state was examined in order to assess the genetic variability conservation.

## 3. Results

In all the goats tested, the total DNA amounts extracted from 200 mL of whole blood and 30–40 hair follicles were enough for the specific amplification of targeted DNA fragments and DNA Sanger sequencing of the *PRNP* gene. This provided high-quality sequences from the two types of biological samples that were easily analysed with BioEdit Sequence Alignment Editor, ver. 7.2.5 (Tom Hall, Ibis biosciences, Carlsbad, CA, USA). *PRNP* polymorphisms were evaluated for 15 codons in 50 Carpathian, 53 Banat’s White, and 53 French Alpine breed goats (Table 4).

In the Carpathian breed, genetic variation was observed at codons 110, 142, 222, and 240; in Banat’s White, at codons 127, 143, 168, 211, 222, and 240; and in French Alpine, at codons 110, 142, 154, 211, 222, and 240. In total, genetic variation that generates amino acid substitutions was observed in nine codons.

The 19 haplotypes identified were not evenly distributed among the three goat breeds studied (Table 5). In this study, the most common haplotype was H6 (62.18%), followed by H7 (17.95%). In Carpathian goats, haplotypes H4 (2.00%), H5 (4.00%), H6 (61,00%), H7 (27.00%), H15 (1.00%), H17 (1.00%), H18 (3.00%), and H19 (1.00%) were detected. In Banat’s White goats, 10 haplotypes were noted, as follows: H2 (0.94%), H4 (7.55%), H6 (71.70%), H7 (5.66%), H8 (1.89%), H9 (3.77%), H11 (2.83%), H12 (2.83%), H13 (1.89%), and H16 (0.94%). The haplotypes detected in French Alpine were H1 (1.89%), H3 (0.94%), H4 (4.72%), H5 (10.38%), H6 (53.77%), H7 (21.70%), H10 (0.94%), H14 (4.72%), and H17 (0.94%). The K222 allele had a frequency of 6% (3/50) in Carpathian, 9.43% (5/53) in Banat’s White, and 15.09% (8/53) in French Alpine. Silent mutations were observed in 49.36% (77/156) of goats (Figure S2), of which 64% (32/50) affected Carpathian goats, 41.51% (22/53) Banat’s White, and 43.40% (23/53) French Alpine (Table S1).

In our work, a sample taken from a goat with classical scrapie was used for *PRNP* sequencing. The scrapie-positive Banat’s White goat (GenBank ID: ON015441) had G37G, W102W, T110T, G127G, I142I, H143H, N146N, R151R, R154R, P168P, S173S, R211R, I218I, Q222Q, and S240S. Following the EFSA summary of evidence on caprine *PRNP* gene alleles associated with susceptibility to TSEs [37] (pp. 30–31), we prepared a comparative table of amino acid polymorphism of our scrapie-positive Banat’s White goat with caprine *PRNP* gene alleles associated with TSE susceptibility previously reported (Table 6). At codon 146, all the goats were homozygote NN. At codon 222, 139 goats were homozygote QQ, 16 homozygote KK, and one goat heterozygote HQ.

## 4. Discussion

Initially validated by Gurău et al. (2021), genomic DNA isolation from hair follicles proved to be, in the case of the 156 goats included in this study, a method with performance similar to the method in which the necessary DNA isolation is made from whole blood samples collected on K3-EDTA. Consequently, the recommendation of this sampling method in selective goat rearing programs to increase scrapie resistance should be considered. Moreover, hair follicles can be sampled from goats by trained farm workers and the materials required for individual sampling and packaging of the sample are inexpensive.

The scientific knowledge concerning genetic resistance to classical scrapie and polymorphisms of the *PRNP* gene in European goat breeds/populations is sufficient to consider the development of guidance on how to increase goat genetic resistance to classical scrapie by disseminating resistant alleles in goat breeds [15,19,26,28,35,37,44,48,49]; our data give particular information for a future Romanian guide of breeding goats resistant to scrapie. Moreover, our research covered two local breeds (a total of 103 GenBank IDs of Carpathian and Banat’s White were obtained) and one of the most common European goat breeds (French Alpine); it also generated data for future metanalyses.

In 2017, the EFSA Panel on Biological Hazards concluded that “the field and experimental data available for the K222, the D146 and the S146 alleles are greater than those available in the public domain for the ARR allele in sheep when the 2002 SSC opinion on safe sourcing of small ruminant materials was produced” [37].

The scrapie-positive goat analysed in our study was a female Banat’s White breed (GenBank ID: ON015441) without polymorphism associated with increased resistance (G37G, W102W, T110T, G127G, I142I, H143H, N146N, R151R, R154R, P168P, S173S, R211R, I218I, Q222Q, and S240S). The genotype ON015441 was consistent with our current understanding of which genotypes are susceptible [15,16,17,22,26,30,31,32,33,37,57,58].

The D146 and the S146 alleles were not detected in the analysed goats.

Unfortunately, the K222 allele was rare in all the analysed Romanian herds. Only 16 homozygote K222K goats were detected in our study, which included: two females and one male in Carpathian goats; three females and two males in Banat’s White goats; and seven females and one male in French Alpine goats. In our study, 89.10% (139/156) of the goats were homozygote Q222Q. One female of the French Alpine breed was heterozygote H222Q.

In Romania, selection plans for scrapie resistance in goats have not been developed due to the lack of information on the genetic resistance of goats to TSEs. Therefore, the *PRNP* polymorphisms, alleles, and haplotypes identified and analysed in our study could contribute to the development of breeding programs to reduce the possible risk of TSEs in goats. Extending the survey to more goats and encouraging genotyping by providing subsidies to farmers may be a future direction for stakeholders. The best start for a future breeding program can be the use of homozygous K222 male goats.

## 5. Conclusions 

Our work highlights that only 9 of 15 analysed codons were with non-synonymous polymorphisms (110, 127, 142, 143, 154, 168, 211, 222, and 240); the frequencies of SNPs were different and not all were present in all three goat breeds. The codons without polymorphisms were G37G, W102W, N146N, R151R, S173S, and I218I. The following non-synonymous polymorphisms of *PRNP* were recorded: P110P, P110S, P110T, T110T, G127G, G127S, I142I, I142M, T142I, H143H, P143P, R143R, R154R, H154R, P168P, Q168Q, Q211Q, Q211R, Q222Q, H222Q, K222K, S240S, P240P, P240S, and S240P. The scrapie-positive Banat’s White goat had G37G, W102W, T110T, G127G, I142I, H143H, N146N, R151R, R154R, P168P, S173S, R211R, I218I, Q222Q, and S240S. The K222 allele has a frequency of 6% (3/50) in Carpathian, 9.43% (5/53) in Banat’s White, and 15.09% (8/53) in French Alpine. Therefore, the polymorphisms detected in our goat breeds are too rare to design a breeding program at the current time. With the support of governments or breeders’ associations, farmers should be encouraged to breed goats for resistance to scrapie mainly through selective reproduction using male goats with scrapie-resistant genotypes. There is also a need for a more extensive study covering more breeds and more farms.

## Figures and Tables

**Table 1:** Sampling, localities, sex, and sample size of the goat breeds studied.

Breed	Localities, County	Number of Animals		
		Males	Females	n *
Carpathian	Cuza Voda, Braila	9	9	18
	Stancuta, Braila	5	27	32
Total Carpathian		14	36	50
Banat’s White	Ion Neculce, Iasi	5	47	52
	Romanu, Braila	0	1	1
*Total* *Banat’s White*		*5*	*48*	*53*
French Alpine	Dudescu, Braila	5	0	5
	Gara Banca, Vaslui	10	38	48
*Total* *French Alpine*		15	38	53
Total		34	122	156

* *n* = total number of goats studied.

**Table 2:** Forward and reverse primers used for PCR amplification of the goat *PRNP* gene [54,55].

Primer	Sequence	Genome Position
PCR amplification		
F1	5′-CATTTATGACCTAGAATGTTTATAGCTGATGCCA-3′	*PRNP* CDS ORF 1200 bp
R1	5′-TTGAATGAATATTATGTGGCCTCCTTCCAGAC-3′	*PRNP* CDS ORF 1200 bp

**Table 3:** Forward and reverse primers used for Sanger sequencing of the goat *PRNP* gene [54,55].

Primer	Sequence	Genome Position
Sanger sequencing		
T3	5′-TTTACGTGGGCATTTGATGC-3′	ORF
T4	5′-GGCTGCAGGTAGACACTCTC-3′	ORF

**Table 4:** The percentages of *PRNP* polymorphisms at 15 codons in 50 Carpathian, 53 Banat’s White, and 53 French Alpine breed goats. The percentages of goats that possess the amino acid specified for each codon position are presented for both homozygote and heterozygote genotypes.

Codon Position	Amino Acid *	Breeds % (*n*/N) *		
		Carpathian	Banat’s White	French Alpine
37	G	100 (50/50)	100 (53/53)	100 (53/53)
102	W	100 (50/50)	100 (53/53)	100 (53/53)
110	P	6 (3/50)	-	1.88 (1/53)
	S	2 (1/50)	-	-
	T	96 (48/50)	100 (53/53)	100 (53/53)
127	G	100 (50/50)	100 (53/53)	100 (53/53)
	S	-	(1/53)	-
142	I	100 (50/50)	100 (53/53)	100 (53/53)
	T	2 (1/50)	-	-
	M	-	-	9.43 (5/53)
143	H	100 (50/50)	86.79 (46/53)	100 (53/53)
	P	-	3.77 (2/53)	-
	R	-	3.77 (2/53)	-
146	N	100 (50/50)	100 (53/53)	100 (53/53)
151	R	100 (50/50)	100 (53/53)	100 (53/53)
154	H	-	-	1.88 (1/53)
	R	100 (50/50)	100 (53/53)	100 (53/53)
168	P	100 (50/50)	86.79 (46/53)	100 (50/50)
	Q	-	7.54 (4/53)	-
173	S	100 (50/50)	100 (53/53)	100 (53/53)
211	Q	-	100 (53/53)	5.66 (3/53)
	R	100 (50/50)	1.88 (1/53)	100 (53/53)
218	I	100 (50/50)	100 (53/53)	100 (53/53)
222	H	-	-	1.88 (1/53)
	Q	94 (47/50)	86.79 (46/53)	84.90 (45/53)
	K	6 (3/50)	7.54 (4/53)	15.09(8/53)
240	P	84 (42/50)	98.11 (52/53)	81.12 (43/53)
	S	56 (28/50)	11.32 (6/53)	50.94 (27/53)

* One conventional letter was used to code the amino acids (e.g., tryptophan: W) in accordance with the IUPAC nomenclature [56]. The number of animals is reported in brackets. *n*/N = the number of animals detected in the population with the specified amino acid/the total number of analysed animals in each breed. -: not detected in breed.

**Table 5:** The percentages of *PRNP* haplotypes at codons 110, 127, 142, 143, 154, 168, 211, 222, and 240 in 50 Carpathian, 53 Banat’s White, and 53 French Alpine breed goats.

	Amino Acid Position									Haplotype Percentage (%)			
Haplotype	110	127	142	143	154	168	211	222	240	Carpathian	Banat’s White	French Alpine	All Goats
1	T	G	I	H	R	P	Q	Q	S	0.00	0.00	1.89	0.64
2	-	-	-	-	-	-	-	-	P	0.00	0.94	0.00	0.32
3	-	-	-	-	-	-	-	H	-	0.00	0.00	0.94	0.32
4	-	-	-	-	-	-	R	K	P	2.00	7.55	4.72	4.81
5	-	-	-	-	-	-	R	K	-	4.00	0.00	10.38	4.81
6	-	-	-	-	-	-	R	-	P	61.00	71.70	53.77	62.18
7	-	-	-	-	-	-	R	-	-	27.00	5.66	21.70	17.95
8	-	-	-	-	-	Q	R	K	P	0.00	1.89	0.00	0.64
9	-	-	-	-	-	Q	R	-	P	0.00	3.77	0.00	1.28
10	-	-	-	-	H	-	R	-	-	0.00	0.00	0.94	0.32
11	-	-	-	P	-	-	R	-	P	0.00	2.83	0.00	0.96
12	-	-	-	P	-	-	R	-	-	0.00	2.83	0.00	0.96
13	-	-	-	R	-	Q	R	-	P	0.00	1.89	0.00	0.64
14	-	-	M	-	-	-	R	-	P	0.00	0.00	4.72	1.60
15	-	-	T	-	-	-	R	-	-	1.00	0.00	0.00	0.32
16	-	S	-	-	-	-	R	-	P	0.00	0.94	0.00	0.32
17	P	-	-	-	-	-	R	-	P	1.00	0.00	0.94	0.64
18	P	-	-	-	-	-	R	-	-	3.00	0.00	0.00	0.96
19	S	-	-	-	-	-	R	-	-	1.00	0.00	0.00	0.32

-: Indicates no amino acid change with respect to haplotype 1.

**Table 6:** Comparative analyses of a classical scrapie-positive Banat’s White goat (GenBank ID: ON015441) amino acid polymorphisms with *PRNP* gene alleles associated with TSE susceptibility previously reported.

*PRNP* Gene Polymorphisms	Codon						
	127	142	143	146	154	211	222
ON015441 *	G–G	I–I	H–H	N–N	R–R	R–R	Q–Q
Amino acid polymorphisms	G–S	I–M	H–R	N–S/D	R–H	R–Q	Q–K
aa *	S	M	R	S/D	H	Q	K
References	[33,42,43,44,45,46]	[33,42,43,44, 47,48,49, 51,52,53]	[33,43,49, 53]	[33,49,50, 53]	[33, 42,43,44,47,49, 50,52,54]	[33,42,44, 45,47,52, 54]	[33,31,33, 42,43,44,45,47,52, 54]

* ON015441: GenBank ID of scrapie-positive Banat’s White goat. Aa: amino acid associated with resistance.

## Data Availability

Not applicable.

## Supplementary Materials

The following supporting information can be downloaded at: https://www.mdpi.com/article/10.3390/genes13081316/s1, Figure S1: Electropherograms showing the polymorphism of the prion protein gene (*PRNP*) identified in Romanian goats by using BioEdit Sequence Alignment Editor (ver. 7.2.5); Figure S2: Electropherograms of three goats with silent mutations; and Table S1: Goats with silent mutations and homo- or heterozygous polymorphisms at 15 codons of the *PRNP* gene.

## References

[1] Prusiner S.B. (1994). Molecular biology and genetics of prion diseases. Philos. Trans. R. Soc. Lond. B Biol. Sci..

[2] Alarcon P., Marco-Jimenez F., Arnold M., Wolf A., Rajanayagam B., Stevens K.B., Adkin A. (2018). Spatio-temporal and risk factor analysis of alleles related to Scrapie resistance in sheep in Great Britain before. during and after a national breeding program. Prev. Vet. Med..

[3] Consolidated Text: Regulation (EC) No 999/2001 of the European Parliament and of the Council of 22 May 2001 Laying down Rules for the Prevention. Control and Eradication of Certain Transmissible Spongiform Encephalopathies. http://eur-lex.europa.eu/legal-content/EN/TXT/?qid=1519030609850&uri=CELEX:02001R0999-20170701.

[4] Erdtmann R., Sivitz L., Institute of Medicine (US) Committee on Transmissible Spongiform Encephalopathies: Assessment of Relevant Science (2003). Advancing Prion Science: Guidance for the National Prion Research Program.

[5] Kellar J.A., Lees V.W. (2003). Risk management of the transmissible spongiform encephalopathies in North America. Rev. Sci. Tech..

[6] Melchior M.B., Windig J.J., Hagenaars T.J., Bossers A., Davidse A., van Zijderveld F.G. (2010). Eradication of scrapie with selective breeding: Are we nearly there?. BMC Vet. Res..

[7] Nicholas F.W. (2005). Animal breeding and disease. Philos. Trans. R. Soc. Lond. B Biol. Sci..

[8] Nodelijk G., van Roermund H.J., van Keulen L.J., Engel B., Vellema P., Hagenaars T.J. (2011). Breeding with resistant rams leads to rapid control of classical scrapie in affected sheep flocks. BMC Vet. Res..

[9] Zabavnik J., Cotman M., Juntes P., Ambrozic I. (2018). A decade of using small-to-medium throughput allele discrimination assay to determine prion protein gene (*Prnp*) genotypes in sheep in Slovenia. J. Vet. Diagn. Investig..

[10] Hunter N., Cairns D., Foster J.D., Smith G., Goldmann W., Donnelly K. (1997). Is scrapie solely a genetic disease?. Nature.

[11] Djaout A., Chiappini B., Gaouar S., Afri-Bouzebda F., Conte M., Chekkal F., El-Bouyahiaoui R., Boukhari R., Agrimi U., Vaccari G. (2018). Biodiversity and selection for scrapie resistance in sheep: Genetic polymorphism in eight breeds of Algeria. J. Genet..

[12] Srithayakumar V., Mitchell G.B., White B.N. (2016). Identification of amino acid variation in the prion protein associated with classical scrapie in Canadian dairy goats. BMC Vet. Res..

[13] Teferedegn E.Y., Yaman Y., Ün C. (2020). Novel Variations in Native Ethiopian Goat breeds *PRNP* Gene and Their Potential Effect on Prion Protein Stability. Sci. Rep..

[14] Vitale M., Migliore S., Tilahun B., Abdurahaman M., Tolone M., Sammarco I., Di Marco Lo Presti V., Gebremedhin E.Z. (2019). Two novel amino acid substitutions in highly conserved regions of prion protein (PrP) and a high frequency of a scrapie protective variant in native Ethiopian goats. BMC Vet. Res..

[15] Gelasakis A.I., Boukouvala E., Babetsa M., Katharopoulos E., Palaska V., Papakostaki D., Giadinis N.D., Loukovitis D., Langeveld J., Ekateriniadou L.V. (2021). Polymorphisms of Codons 110, 146, 211 and 222 at the Goat *PRNP* Locus and Their Association with Scrapie in Greece. Animals.

[16] Kanata E., Humphreys-Panagiotidis C., Giadinis N.D., Papaioannou N., Arsenakis M., Sklaviadis T. (2014). Perspectives of a scrapie resistance breeding scheme targeting Q211, S146 and K222 caprine *PRNP* alleles in Greek goats. Vet. Res..

[17] Vouraki S., Gelasakis A.I., Alexandri P., Boukouvala E., Ekateriniadou L.V., Banos G., Arsenos G. (2018). Genetic profile of scrapie codons 146, 211 and 222 in the *PRNP* gene locus in three breeds of dairy goats. PLoS ONE.

[18] Migliore S., Agnello S., D’Avola S., Goldmann W., Di Marco Lo Presti V., Vitale M. (2017). A cross-sectional study of *PRNP* gene in two native Sicilian goat populations in Italy: A relation between prion gene polymorphisms and scrapie incidence. J. Genet..

[19] Torricelli M., Sebastiani C., Ciullo M., Ceccobelli S., Chiappini B., Vaccari G., Capocefalo A., Conte M., Giovannini S., Lasagna E. (2021). *PRNP* polymorphisms in eight local goat populations/breeds from central and southern Italy. Animals.

[20] Vitale M., Migliore S., La Giglia M., Alberti P., Di Marco Lo Presti V., Langeveld J.P. (2016). Scrapie incidence and *PRNP* polymorphisms: Rare small ruminant breeds of Sicily with TSE protecting genetic reservoirs. BMC Vet. Res..

[21] Jeong M.J., Kim Y.C., Jeong B.H. (2018). Prion-like protein gene (PRND) polymorphisms associated with scrapie susceptibility in Korean native black goats. PLoS ONE.

[22] Kim S.K., Kim Y.C., Won S.Y., Jeong B.H. (2019). Potential scrapie-associated polymorphisms of the prion protein gene (*PRNP*) in Korean native black goats. Sci. Rep..

[23] Acín C., Martín-Burriel I., Monleón E., Lyahyai J., Pitarch J.L., Serrano C., Monzón M., Zaragoza P., Badiola J.J. (2013). Prion protein gene variability in Spanish goats. Inference through susceptibility to classical scrapie strains and pathogenic distribution of peripheral PrP(sc.). PLoS ONE.

[24] Pitarch J.L., Raksa H.C., Arnal M.C., Revilla M., Martínez D., Fernández de Luco D., Badiola J.J., Goldmann W., Acín C. (2018). Low sequence diversity of the prion protein gene (*PRNP*) in wild deer and goat species from Spain. Vet. Res..

[25] Meydan H., Pehlivan E., Özkan M.M., Yildiz M.A., Goldmann W. (2017). Prion protein gene polymorphisms in Turkish native goat breeds. J. Genet..

[26] Kdidi S., Yahyaoui M.H., Conte M., Chiappini B., Hammadi M., Khorchani T., Vaccari G. (2021). Genetic variation in the prion protein gene (*PRNP*) of two Tunisian goat populations. Animals.

[27] Goldmann W., Marier E., Stewart P., Konold T., Street S., Langeveld J., Windl O., Ortiz-Pelaez A. (2016). Prion protein genotype survey confirms low frequency of scrapie-resistant K222 allele in British goat herds. Vet. Rec..

[28] Zeineldin M., Lehman K., Urie N., Branan M., Wiedenheft A., Marshall K., Robbe-Austerman S., Thacker T. (2021). Large-scale survey of prion protein genetic variability in scrapie disease-free goats from the United States. PLoS ONE.

[29] Acutis P.L., Martucci F., D’Angelo A., Peletto S., Colussi S., Maurella C., Porcario C., Iulini B., Mazza M., Dell’Atti L. (2012). Resistance to classical scrapie in experimentally challenged goats carrying mutation K222 of the prion protein gene. Vet. Res..

[30] Barillet F., Mariat D., Amigues Y., Faugeras R., Caillat H., Moazami-Goudarzi K., Rupp R., Babillìot J.M., Lacroux C., Lugan S. (2009). Identification of seven haplotypes of the caprine PrP gene at codons 127, 142, 154, 211, 222, and 240 in French Alpine and Saanen breeds and their association with classical scrapie. J. Gen. Virol..

[31] Bouzalas I.G., Dovas C., Banos G., Papanastasopoulou M., Kritas S., Oevermann A., Papakostaki D., Evangelia C., Papadopoulos O., Seuberlich T. (2010). Caprine *PRNP* polymorphisms at codons 171. 211. 222 and 240 in a Greek herd and their association with classical scrapie. J. Gen. Virol..

[32] Fast C., Goldmann W., Berthon P., Tauscher K., Andréoletti O., Lantier I., Rossignol C., Bossers A., Jacobs J.G., Hunter N. (2017). Protecting effect of PrP codons M142 and K222 in goats orally challenged with bovine spongiform encephalopathy prions. Vet. Res..

[33] Fragkiadaki E.G., Vaccari G., Ekateriniadou L.V., Agrimi U., Giadinis N.D., Chiappini B., Esposito E., Conte M., Nonno R. (2011). *PRNP* genetic variability and molecular typing of natural goat scrapie isolates in a high number of infected herds. Vet. Res..

[34] Lacroux C., Perrin-Chauvineau C., Corbière F., Aron N., Aguilar-Calvo P., Torres J.M., Costes P., Brémaud I., Lugan S., Schelcher F. (2014). Genetic resistance to scrapie infection in experimentally challenged goats. J. Virol..

[35] Madsen-Bouterse S.A., Stewart P., Williamson H., Schneider D.A., Goldmann W. (2021). Caprine *PRNP* polymorphisms N146S and Q222K are associated with proteolytic cleavage of PrPC. Genet. Sel. Evol..

[36] Vaccari G., Di Bari M.A., Morelli L., Nonno R., Chiappini B., Antonucci G., Marcon S., Esposito E., Fazzi P., Palazzini N. (2006). Identification of an allelic variant of the goat PrP gene associated with resistance to scrapie. J. Gen. Virol..

[37] Ricci A., Allende A., Bolton D., Chemaly M., Davies R., Fernandez Escamez P.S., Girones R., Herman L., Koutsoumanis K., European Food Safety Authority (EFSA) BIOHAZ Panel (EFSA Panel on Biological Hazards) (2017). Scientific opinion on genetic resistance to transmissible spongiform encephalopathies (TSE) in goats. EFSA J..

[38] European Food Safety Authority (EFSA) (2019). The European Union summary report on surveillance for the presence of transmissible spongiform encephalopathies (TSE) in 2018. EFSA J..

[39] European Food Safety Authority (EFSA) (2020). The European Union summary report on surveillance for the presence of transmissible spongiform encephalopathies (TSE) in 2019. EFSA J..

[40] European Food Safety Authority (EFSA) (2021). The European Union summary report on surveillance for the presence of transmissible spongiform encephalopathies (TSE) in 2020. EFSA J..

[41] Arnold M., Ru G., Simmons M., Vidal-Diez A., Ortiz-Pelaez A., Stella P., European Food Safety Authority (EFSA) (2021). Scientific report on the analysis of the 2-year compulsory intensified monitoring of atypical scrapie. EFSA J..

[42] Kusza S., Ilie D.E., Sauer M., Nagy K., Patras I., Gavojdian D. (2016). Genetic polymorphism of CSN2 gene in Banat White and Carpatina goats. Acta Biochim. Pol..

[43] Călin I., Răducută I., Dărăban S., Vlad I., Priseceanu H.I., Pascal C., Pădeanu I. (2015). Research on quantitative skills in meat production direction at youth goats from Carpathian breed in relation with the rearing system. Agric. Agric. Sci. Procedia.

[44] Migdał W., Kawęcka A., Sikora J., Migdał Ł. (2021). Meat Quality of the Native Carpathian Goat Breed in Comparison with the Saanen Breed. Animals.

[45] Padeanu I. (2001). Sheep and Goat Breeding Technology (Tehnologia Cresterii Ovinelor si Caprinelor).

[46] Pascal C., Padeanu I., Calin I., Daraban S., Nacu G. (2011). Researches related to meat yield aptitudes of Carpatina breed reared in Romania. Sci. Pap. Anim. Sci. Biotech..

[47] Voia S., Padeanu I., Daraban S., Mot T., Dronca D., Pet I., Gavojdian D., Ivan M. (2010). Study regarding goat milk composition and the growth rate in kids of Carpatina goat breed. Sci. Pap. Anim. Sci. Biotech..

[48] Shuvarikov A.S., Pastukh O.N., Zhukova E.V., Zheltova O.A. (2021). The quality of milk of goats of Saanen, Alpine and Nubian breeds. IOP Conf. Ser. Earth Environ. Sci..

[49] Duricic D., Zaja I.Z., Benic M., Sukalic T., Kovacic M., Samardzija M. (2021). Relationship between reproductive performance and meteorological variables in French Alpine goats in the northwestern part of Croatia. Anim. Behav. Biometeorol..

[50] Gurău M.R., Crețu D.M., Negru E., Ionescu T., Udriste A.A., Cornea P., Bărăităreanu S. (2021). Comparative analysis of total DNA isolation procedure from blood and hair follicle samples in goats. Rev. Romana Med. Vet..

[51] PureLink Genomic DNA Kits. https://tools.thermofisher.com/content/sfs/manuals/purelink_genomic_man.pdf.

[52] Qubit™ dsDNA HS Assay Kit. https://assets.thermofisher.com/TFS-Assets/LSG/manuals/Qubit_dsDNA_HS_Assay_UG.pdf.

[53] Qubit™ dsDNA HS and BR Assay Kits. https://www.thermofisher.com/order/catalog/product/Q32851.

[54] Migliore S., Agnello S., Chiappini B., Vaccari G., Mignacca S.A., Di Marco Lo Presti V., Di Domenico F., Vitale M. (2015). Biodiversity and selection for scrapie resistance in goats: Genetic polymorphism in “Girgentana” breed in Sicily, Italy. Small Rumin. Res..

[55] Vaccari G., Panagiotidis C.H., Acin C., Peletto S., Barillet F., Acutis P. (2009). State-of-the-art review of goat TSE in the European Union, with special emphasis on *PRNP* genetics and epidemiology. Vet. Res..

[56] (1984). IUPAC-IUB Joint Commission on Biochemical Nomenclature Nomenclature and Symbolism for Amino Acids and Peptides. Eur. J. Biochem..

[57] Billinis C., Panagiotidis C.H., Psychas V., Argyroudis S., Nicolaou A., Leontides S., Papadopoulos O., Sklaviadis T. (2002). Prion protein gene polymorphisms in natural goat scrapie. J. Gen. Virol..

[58] Acutis P.L., Bossers A., Priem J., Riina M.V., Peletto S., Mazza M., Casalone C., Forloni G., Ru G., Caramelli M. (2006). Identification of prion protein gene polymorphisms in goats from Italian scrapie outbreaks. J. Gen. Virol..

