# Supplementary material for: Genetic Polymorphism at 15 Codons of the Prion Protein Gene in 156 Goats from Romania
Source: Genes (Basel). 2022 Jul 23;13(8):1316. doi: 10.3390/genes13081316 (PMC9394368; doi:10.3390/genes13081316)
Supplement: Supplementary file 1 [file genes-13-01316-s001.zip › Figure S1.pdf]

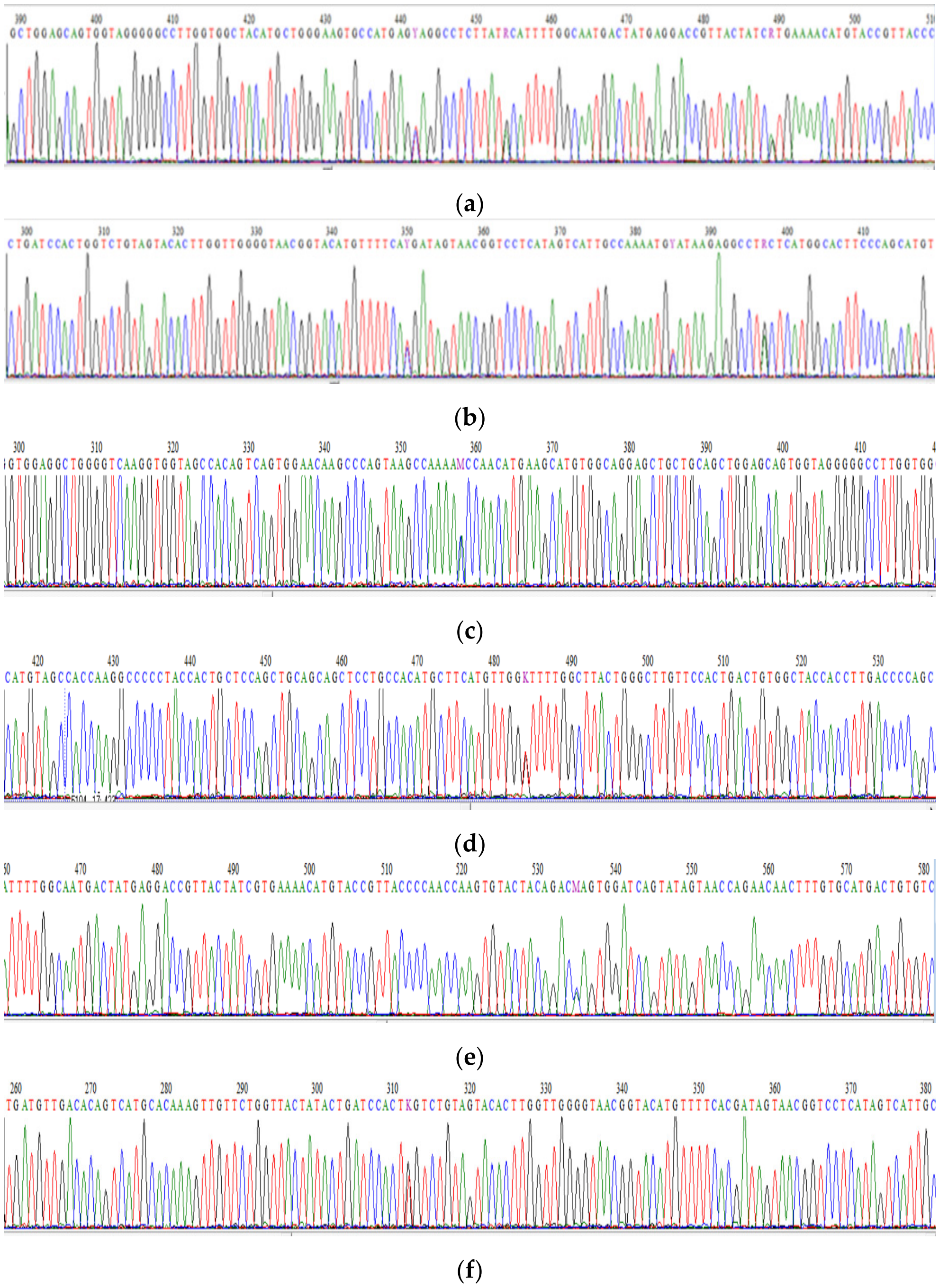

Figure 1. Electropherograms showing the polymorphism of the prion protein gene (*PRNP*) identified in Romanian goats by using BioEdit Sequence Alignment Editor (ver. 7.2.5). (**a**) PRNP polymorphism on forward at codons I142M (G C/T A), H154R (T A/G C), R211R (C A/G T), French Alpine (GenBank ID: ON084837); (**b**) PRNP polymorphism on reverse at codons I142M (G G/A T), H154R (A T/C G), R211R (G T/C A), French Alpine (GenBank ID: ON084837); (**c**) PRNP polymorphism on forward at codon P110S (A C/A C), Carpathian (GenBank ID: ON417730); (**d**) PRNP polymorphism on reverse at codon P110S (G G/T T), Carpathian (GenBank ID: ON417730); (**e**) PRNP polymorphism on forward at codon Q168Q (C A/C A), Banat's White (GenBank ID: ON084819); (**f**) PRNP polymorphism on reverse at codon Q168Q (T G/T G), Banat's White (GenBank ID: ON084819). The colours (green: adenine; blue: cytosine; black: guanine; red: thymine) indicate individual bases of DNA sequence using an automatic sequencer ABI PRISM 3130 (Applied Biosystems).
