# Supplementary material for: Genetic Polymorphism at 15 Codons of the Prion Protein Gene in 156 Goats from Romania
Source: Genes (Basel). 2022 Jul 23;13(8):1316. doi: 10.3390/genes13081316 (PMC9394368; doi:10.3390/genes13081316)
Supplement: Supplementary file 1 [file genes-13-01316-s001.zip › Figure S2.pdf]

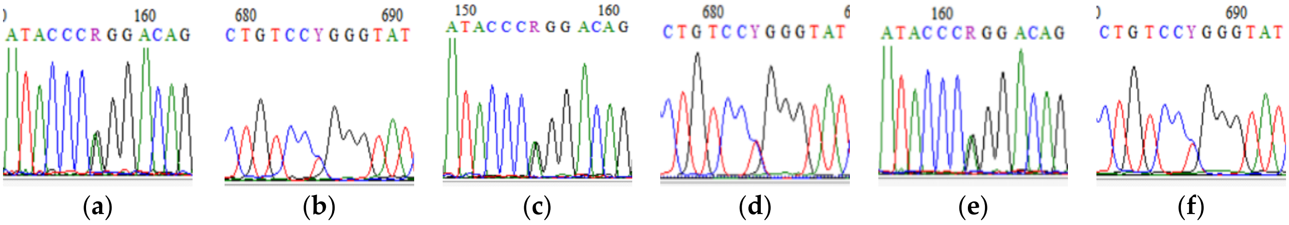

Figure S2. Electropherograms of three goats with silent mutations. (**a**) codon W34W in the 5′ UTR with adenine silent mutation (GenBank ID: ON015426); (**b**) codon W34W in the 3′ UTR with thymine silent mutation (GenBank ID: ON015426); (**c**) codon W34W in the 5′ UTR with adenine silent mutation (GenBank ID: ON015429); (**d**) codon W34W in the 3′ UTR with thymine silent mutation (GenBank ID: ON015429); (**e**) codon W34W in the 5′ UTR with adenine silent mutation (GenBank ID: ON015440); (**f**) codon W34W in the 3′ UTR with thymine silent mutation (GenBank ID: ON015440). The colours (green: adenine; blue: cytosine; black: guanine; red: thymine) indicate individual bases of DNA sequence using an automatic sequencer ABI PRISM 3130 (Applied Biosystems).
