# Supplementary material for: Genetic Polymorphism at 15 Codons of the Prion Protein Gene in 156 Goats from Romania
Source: Genes (Basel). 2022 Jul 23;13(8):1316. doi: 10.3390/genes13081316 (PMC9394368; doi:10.3390/genes13081316)
Supplement: Supplementary file 1 [file genes-13-01316-s001.zip › Table S1.pdf]

Table S1. Goats with silent mutations and homo- or heterozygous polymorphisms at 15 codons of PRNP gene

#	Code number	Breed	Sex	Age (years)	Silent mutations	37	102	110	127	142	143	146	151	154	168	173	211	218	222	240	GenBank ID
1.	1C	Carpathian	M	4	Yes	G/G	W/W	T/T	G/G	I/I	H/H	N/N	R/R	R/R	P/P	S/S	R/R	I/I	Q/Q	P/P	OM988074
2.	2C	Carpathian	M	4		G/G	W/W	T/T	G/G	I/I	H/H	N/N	R/R	R/R	P/P	S/S	R/R	I/I	Q/Q	P/P	OM988076
3.	3C	Carpathian	M	4	Yes	G/G	W/W	T/T	G/G	I/I	H/H	N/N	R/R	R/R	P/P	S/S	R/R	I/I	Q/Q	S/P	ON417708
4.	4C	Carpathian	M	4	Yes	G/G	W/W	T/T	G/G	I/I	H/H	N/N	R/R	R/R	P/P	S/S	R/R	I/I	Q/Q	S/P	ON417709
5.	5C	Carpathian	M	4		G/G	W/W	T/T	G/G	I/I	H/H	N/N	R/R	R/R	P/P	S/S	R/R	I/I	Q/Q	P/P	ON417710
6.	6C	Carpathian	M	4	Yes	G/G	W/W	T/T	G/G	I/I	H/H	N/N	R/R	R/R	P/P	S/S	R/R	I/I	Q/Q	S/S	ON417711
7.	7C	Carpathian	M	4		G/G	W/W	T/T	G/G	I/I	H/H	N/N	R/R	R/R	P/P	S/S	R/R	I/I	Q/Q	S/P	ON417712
8.	8C	Carpathian	M	4		G/G	W/W	T/T	G/G	I/I	H/H	N/N	R/R	R/R	P/P	S/S	R/R	I/I	Q/Q	P/P	ON417713
9.	9C	Carpathian	M	4		G/G	W/W	T/T	G/G	I/I	H/H	N/N	R/R	R/R	P/P	S/S	R/R	I/I	Q/Q	P/P	ON417714
10.	10C	Carpathian	F	5	Yes	G/G	W/W	T/T	G/G	I/I	H/H	N/N	R/R	R/R	P/P	S/S	R/R	I/I	Q/Q	P/S	OM988073
11.	12C	Carpathian	F	4	Yes	G/G	W/W	T/T	G/G	I/I	H/H	N/N	R/R	R/R	P/P	S/S	R/R	I/I	Q/Q	S/P	ON417715
12.	13C	Carpathian	F	7	Yes	G/G	W/W	T/T	G/G	I/I	H/H	N/N	R/R	R/R	P/P	S/S	R/R	I/I	Q/Q	P/P	ON417716
13.	14C	Carpathian	F	7	Yes	G/G	W/W	T/T	G/G	I/I	H/H	N/N	R/R	R/R	P/P	S/S	R/R	I/I	Q/Q	S/S	ON417717
14.	15C	Carpathian	F	9	Yes	G/G	W/W	T/T	G/G	I/I	H/H	N/N	R/R	R/R	P/P	S/S	R/R	I/I	Q/Q	P/P	ON417718
15.	16C	Carpathian	F	4		G/G	W/W	T/T	G/G	I/I	H/H	N/N	R/R	R/R	P/P	S/S	R/R	I/I	Q/Q	P/P	ON417719
16.	17C	Carpathian	F	7		G/G	W/W	T/T	G/G	I/I	H/H	N/N	R/R	R/R	P/P	S/S	R/R	I/I	Q/Q	P/P	ON417720
17.	18C	Carpathian	F	4	Yes	G/G	W/W	T/T	G/G	I/I	H/H	N/N	R/R	R/R	P/P	S/S	R/R	I/I	Q/Q	P/P	ON417721
18.	19C	Carpathian	F	4	Yes	G/G	W/W	T/T	G/G	I/I	H/H	N/N	R/R	R/R	P/P	S/S	R/R	I/I	Q/Q	P/P	ON417722
19.	20C	Carpathian	F	7	Yes	G/G	W/W	T/T	G/G	I/I	H/H	N/N	R/R	R/R	P/P	S/S	R/R	I/I	Q/Q	P/P	ON417723
20.	21C	Carpathian	F	5		G/G	W/W	T/T	G/G	I/I	H/H	N/N	R/R	R/R	P/P	S/S	R/R	I/I	Q/Q	P/P	ON417724
21.	22C	Carpathian	F	7	Yes	G/G	W/W	T/T	G/G	I/I	H/H	N/N	R/R	R/R	P/P	S/S	R/R	I/I	Q/Q	S/P	ON417725
22.	23C	Carpathian	F	3		G/G	W/W	T/T	G/G	I/I	H/H	N/N	R/R	R/R	P/P	S/S	R/R	I/I	Q/Q	P/P	ON417726
23.	24C	Carpathian	F	7	Yes	G/G	W/W	T/T	G/G	I/I	H/H	N/N	R/R	R/R	P/P	S/S	R/R	I/I	K/K	S/S	ON417727
24.	25C	Carpathian	F	6		G/G	W/W	T/T	G/G	I/I	H/H	N/N	R/R	R/R	P/P	S/S	R/R	I/I	Q/Q	P/P	ON417728
25.	26C	Carpathian	M	4	Yes	G/G	W/W	P/P	G/G	I/I	H/H	N/N	R/R	R/R	P/P	S/S	R/R	I/I	Q/Q	S/P	ON417729
26.	27C	Carpathian	M	4	Yes	G/G	W/W	P/S	G/G	I/I	H/H	N/N	R/R	R/R	P/P	S/S	R/R	I/I	Q/Q	S/S	ON417730

#	Code number	Breed	Sex	Age (years)	Silent mutations	37	102	110	127	142	143	146	151	154	168	173	211	218	222	240	GenBank ID
27.	28C	Carpathian	M	4	Yes	G/G	W/W	T/T	G/G	I/I	H/H	N/N	R/R	R/R	P/P	S/S	R/R	I/I	K/K	S/P	ON417731
28.	29C	Carpathian	M	4		G/G	W/W	T/T	G/G	I/I	H/H	N/N	R/R	R/R	P/P	S/S	R/R	I/I	Q/Q	P/P	ON453970
29.	30C	Carpathian	M	4		G/G	W/W	T/T	G/G	I/I	H/H	N/N	R/R	R/R	P/P	S/S	R/R	I/I	Q/Q	P/P	ON453971
30.	31C	Carpathian	F	5	Yes	G/G	W/W	T/T	G/G	I/I	H/H	N/N	R/R	R/R	P/P	S/S	R/R	I/I	Q/Q	S/S	ON453972
31.	32C	Carpathian	F	3	Yes	G/G	W/W	T/T	G/G	I/I	H/H	N/N	R/R	R/R	P/P	S/S	R/R	I/I	Q/Q	S/P	ON453973
32.	33C	Carpathian	F	4	Yes	G/G	W/W	P/T	G/G	I/I	H/H	N/N	R/R	R/R	P/P	S/S	R/R	I/I	Q/Q	S/P	ON453974
33.	34C	Carpathian	F	4	Yes	G/G	W/W	T/T	G/G	I/I	H/H	N/N	R/R	R/R	P/P	S/S	R/R	I/I	Q/Q	S/P	ON453975
34.	35C	Carpathian	F	3	Yes	G/G	W/W	T/T	G/G	I/I	H/H	N/N	R/R	R/R	P/P	S/S	R/R	I/I	Q/Q	S/P	ON453976
35.	36C	Carpathian	F	3	Yes	G/G	W/W	T/T	G/G	I/I	H/H	N/N	R/R	R/R	P/P	S/S	R/R	I/I	Q/Q	S/P	ON453977
36.	37C	Carpathian	F	5		G/G	W/W	T/T	G/G	I/I	H/H	N/N	R/R	R/R	P/P	S/S	R/R	I/I	Q/Q	P/P	ON453978
37.	38C	Carpathian	F	3		G/G	W/W	T/T	G/G	I/I	H/H	N/N	R/R	R/R	P/P	S/S	R/R	I/I	Q/Q	S/S	ON453979
38.	39C	Carpathian	F	3	Yes	G/G	W/W	T/T	G/G	I/I	H/H	N/N	R/R	R/R	P/P	S/S	R/R	I/I	Q/Q	S/S	ON453980
39.	40C	Carpathian	F	3	Yes	G/G	W/W	T/T	G/G	I/I	H/H	N/N	R/R	R/R	P/P	S/S	R/R	I/I	Q/Q	S/P	ON453981
40.	41C	Carpathian	F	3		G/G	W/W	T/T	G/G	I/I	H/H	N/N	R/R	R/R	P/P	S/S	R/R	I/I	Q/Q	P/P	ON453982
41.	42C	Carpathian	F	5	Yes	G/G	W/W	T/T	G/G	I/I	H/H	N/N	R/R	R/R	P/P	S/S	R/R	I/I	Q/Q	S/P	ON453983
42.	43C	Carpathian	F	4		G/G	W/W	T/T	G/G	I/I	H/H	N/N	R/R	R/R	P/P	S/S	R/R	I/I	Q/Q	P/P	ON453984
43.	44C	Carpathian	F	3		G/G	W/W	T/T	G/G	I/I	H/H	N/N	R/R	R/R	P/P	S/S	R/R	I/I	Q/Q	S/P	ON453985
44.	46C	Carpathian	F	4	Yes	G/G	W/W	T/T	G/G	I/I	H/H	N/N	R/R	R/R	P/P	S/S	R/R	I/I	Q/Q	S/P	ON453986
45.	47C	Carpathian	F	3	Yes	G/G	W/W	T/T	G/G	I/I	H/H	N/N	R/R	R/R	P/P	S/S	R/R	I/I	Q/Q	P/P	ON453987
46.	48C	Carpathian	F	3	Yes	G/G	W/W	T/T	G/G	I/I	H/H	N/N	R/R	R/R	P/P	S/S	R/R	I/I	K/K	S/P	ON453988
47.	49C	Carpathian	F	5		G/G	W/W	T/T	G/G	I/I	H/H	N/N	R/R	R/R	P/P	S/S	R/R	I/I	Q/Q	P/P	ON453989
48.	50C	Carpathian	F	3	Yes	G/G	W/W	T/T	G/G	I/I	H/H	N/N	R/R	R/R	P/P	S/S	R/R	I/I	Q/Q	S/P	ON453990
49.	51C	Carpathian	F	6	Yes	G/G	W/W	T/T	G/G	I/I	H/H	N/N	R/R	R/R	P/P	S/S	R/R	I/I	Q/Q	S/S	ON453991
50.	52C	Carpathian	F	3	Yes	G/G	W/W	T/T	G/G	T/I	H/H	N/N	R/R	R/R	P/P	S/S	R/R	I/I	Q/Q	S/P	ON453992
51.	IV-POZ	Banat's White	F	5		G/G	W/W	T/T	G/G	I/I	H/H	N/N	R/R	R/R	P/P	S/S	R/R	I/I	Q/Q	S/S	ON015441
52.	1 AB	Banat's White	F	1		G/G	W/W	T/T	G/G	I/I	H/H	N/N	R/R	R/R	P/P	S/S	R/R	I/I	Q/Q	P/P	ON149623

#	Code number	Breed	Sex	Age (years)	Silent mutations	37	102	110	127	142	143	146	151	154	168	173	211	218	222	240	GenBank ID
53.	2 AB	Banat's White	F	10		G/G	W/W	T/T	G/G	I/I	H/H	N/N	R/R	R/R	P/P	S/S	R/R	I/I	Q/Q	P/P	ON084850
54.	3 AB	Banat's White	F	2		G/G	W/W	T/T	G/G	I/I	H/H	N/N	R/R	R/R	P/P	S/S	R/R	I/I	Q/Q	P/P	ON084851
55.	4 AB	Banat's White	F	5		G/G	W/W	T/T	G/G	I/I	H/H	N/N	R/R	R/R	P/P	S/S	R/R	I/I	Q/Q	P/P	ON084852
56.	5 AB	Banat's White	F	7	Yes	G/G	W/W	T/T	G/G	I/I	H/H	N/N	R/R	R/R	P/P	S/S	R/R	I/I	Q/Q	S/P	ON084853
57.	6 AB	Banat's White	F	9		G/G	W/W	T/T	G/G	I/I	H/H	N/N	R/R	R/R	P/P	S/S	R/R	I/I	Q/Q	P/P	ON084854
58.	7 AB	Banat's White	M	4	Yes	G/G	W/W	T/T	G/G	I/I	H/H	N/N	R/R	R/R	P/P	S/S	Q/R	I/I	Q/Q	P/P	ON084855
59.	8 AB	Banat's White	F	5		G/G	W/W	T/T	G/G	I/I	H/H	N/N	R/R	R/R	P/P	S/S	R/R	I/I	Q/Q	P/P	ON084856
60.	9 AB	Banat's White	F	5	Yes	G/G	W/W	T/T	G/G	I/I	H/H	N/N	R/R	R/R	P/P	S/S	R/R	I/I	Q/Q	P/P	ON084857
61.	10 AB	Banat's White	F	5	Yes	G/G	W/W	T/T	G/G	I/I	H/H	N/N	R/R	R/R	P/P	S/S	R/R	I/I	Q/Q	P/S	ON084858
62.	11 AB	Banat's White	F	10	Yes	G/G	W/W	T/T	G/G	I/I	P/P	N/N	R/R	R/R	P/P	S/S	R/R	I/I	Q/Q	P/S	ON045072
63.	12 AB	Banat's White	F	5		G/G	W/W	T/T	G/G	I/I	R/R	N/N	R/R	R/R	P/P	S/S	R/R	I/I	Q/Q	P/P	ON084820
64.	13 AB	Banat's White	F	2	Yes	G/G	W/W	T/T	G/G	I/I	H/H	N/N	R/R	R/R	Q/Q	S/S	R/R	I/I	Q/Q	P/P	ON084819
65.	14 AB	Banat's White	F	10		G/G	W/W	T/T	G/G	I/I	P/P	N/N	R/R	R/R	P/P	S/S	R/R	I/I	Q/Q	P/P	ON084818
66.	15 AB	Banat's White	F	9	Yes	G/G	W/W	T/T	G/G	I/I	H/H	N/N	R/R	R/R	P/P	S/S	R/R	I/I	Q/Q	P/P	ON149624
67.	16 AB	Banat's White	M	5		G/G	W/W	T/T	G/G	I/I	H/H	N/N	R/R	R/R	P/P	S/S	R/R	I/I	Q/Q	P/P	ON149625
68.	17 AB	Banat's White	F	2		G/G	W/W	T/T	G/G	I/I	H/H	N/N	R/R	R/R	P/P	S/S	R/R	I/I	Q/Q	P/S	ON149626

#	Code number	Breed	Sex	Age (years)	Silent mutations	37	102	110	127	142	143	146	151	154	168	173	211	218	222	240	GenBank ID
69.	18 AB	Banat's White	F	7		G/G	W/W	T/T	G/G	I/I	H/H	N/N	R/R	R/R	P/P	S/S	R/R	I/I	Q/Q	P/P	ON149627
70.	19 AB	Banat's White	F	2		G/G	W/W	T/T	G/G	I/I	H/H	N/N	R/R	R/R	P/P	S/S	R/R	I/I	Q/Q	P/P	ON149628
71.	20 AB	Banat's White	F	7		G/G	W/W	T/T	G/G	I/I	H/H	N/N	R/R	R/R	P/P	S/S	R/R	I/I	Q/Q	P/P	ON149629
72.	21 AB	Banat's White	F	5		G/G	W/W	T/T	G/S	I/I	H/H	N/N	R/R	R/R	P/P	S/S	R/R	I/I	Q/Q	P/P	ON149630
73.	22 AB	Banat's White	F	7	Yes	G/G	W/W	T/T	G/G	I/I	H/H	N/N	R/R	R/R	P/P	S/S	R/R	I/I	K/K	P/P	ON149631
74.	23 AB	Banat's White	F	1		G/G	W/W	T/T	G/G	I/I	H/H	N/N	R/R	R/R	P/P	S/S	R/R	I/I	Q/Q	P/P	ON149632
75.	24 AB	Banat's White	F	10		G/G	W/W	T/T	G/G	I/I	H/H	N/N	R/R	R/R	P/P	S/S	R/R	I/I	Q/Q	P/P	ON149633
76.	25 AB	Banat's White	F	1		G/G	W/W	T/T	G/G	I/I	H/H	N/N	R/R	R/R	P/P	S/S	R/R	I/I	Q/Q	P/P	ON149634
77.	26 AB	Banat's White	F	2		G/G	W/W	T/T	G/G	I/I	R/R	N/N	R/R	R/R	Q/Q	S/S	R/R	I/I	Q/Q	P/P	ON149635
78.	27 AB	Banat's White	F	5	Yes	G/G	W/W	T/T	G/G	I/I	H/H	N/N	R/R	R/R	P/P	S/S	R/R	I/I	Q/Q	P/P	ON149636
79.	28 AB	Banat's White	F	9	Yes	G/G	W/W	T/T	G/G	I/I	H/H	N/N	R/R	R/R	P/P	S/S	R/R	I/I	Q/Q	P/P	ON149637
80.	29 AB	Banat's White	F	2	Yes	G/G	W/W	T/T	G/G	I/I	H/H	N/N	R/R	R/R	P/P	S/S	R/R	I/I	Q/Q	P/S	ON149638
81.	30 AB	Banat's White	F	1		G/G	W/W	T/T	G/G	I/I	H/H	N/N	R/R	R/R	P/P	S/S	R/R	I/I	Q/Q	P/P	ON149640
82.	31 AB	Banat's White	F	2	Yes	G/G	W/W	T/T	G/G	I/I	H/H	N/N	R/R	R/R	Q/Q	S/S	R/R	I/I	Q/Q	P/P	ON072522
83.	32 AB	Banat's White	F	10	Yes	G/G	W/W	T/T	G/G	I/I	H/H	N/N	R/R	R/R	P/P	S/S	R/R	I/I	Q/Q	P/P	ON149639
84.	33 AB	Banat's White	F	1		G/G	W/W	T/T	G/G	I/I	H/H	N/N	R/R	R/R	P/P	S/S	R/R	I/I	Q/Q	P/P	ON149641

#	Code number	Breed	Sex	Age (years)	Silent mutations	37	102	110	127	142	143	146	151	154	168	173	211	218	222	240	GenBank ID
85.	34 AB	Banat's White	F	2		G/G	W/W	T/T	G/G	I/I	H/H	N/N	R/R	R/R	P/P	S/S	R/R	I/I	Q/Q	P/P	ON149642
86.	35 AB	Banat's White	F	10		G/G	W/W	T/T	G/G	I/I	H/H	N/N	R/R	R/R	P/P	S/S	R/R	I/I	Q/Q	P/P	ON149643
87.	36 AB	Banat's White	F	2		G/G	W/W	T/T	G/G	I/I	H/H	N/N	R/R	R/R	P/P	S/S	R/R	I/I	Q/Q	P/P	ON149644
88.	37 AB	Banat's White	F	2	Yes	G/G	W/W	T/T	G/G	I/I	H/H	N/N	R/R	R/R	P/P	S/S	R/R	I/I	Q/Q	P/P	ON149645
89.	38 AB	Banat's White	M	5	Yes	G/G	W/W	T/T	G/G	I/I	H/H	N/N	R/R	R/R	P/P	S/S	R/R	I/I	K/K	P/P	ON149646
90.	39 AB	Banat's White	F	2		G/G	W/W	T/T	G/G	I/I	H/H	N/N	R/R	R/R	P/P	S/S	R/R	I/I	Q/Q	P/P	ON149647
91.	40 AB	Banat's White	F	7	Yes	G/G	W/W	T/T	G/G	I/I	H/H	N/N	R/R	R/R	Q/Q	S/S	R/R	I/I	K/K	P/P	ON149648
92.	41 AB	Banat's White	F	7	Yes	G/G	W/W	T/T	G/G	I/I	H/H	N/N	R/R	R/R	P/P	S/S	R/R	I/I	Q/Q	P/P	ON149649
93.	42 AB	Banat's White	M	4		G/G	W/W	T/T	G/G	I/I	H/H	N/N	R/R	R/R	P/P	S/S	R/R	I/I	Q/Q	P/P	ON149650
94.	43 AB	Banat's White	F	1	Yes	G/G	W/W	T/T	G/G	I/I	H/H	N/N	R/R	R/R	P/P	S/S	R/R	I/I	K/K	P/P	ON149651
95.	44 AB	Banat's White	M	7	Yes	G/G	W/W	T/T	G/G	I/I	H/H	N/N	R/R	R/R	P/P	S/S	R/R	I/I	K/K	P/P	ON149652
96.	45 AB	Banat's White	F	1		G/G	W/W	T/T	G/G	I/I	H/H	N/N	R/R	R/R	P/P	S/S	R/R	I/I	Q/Q	P/P	ON149653
97.	46 AB	Banat's White	F	2	Yes	G/G	W/W	T/T	G/G	I/I	H/H	N/N	R/R	R/R	P/P	S/S	R/R	I/I	Q/Q	P/P	ON149654
98.	47 AB	Banat's White	F	2		G/G	W/W	T/T	G/G	I/I	H/H	N/N	R/R	R/R	P/P	S/S	R/R	I/I	Q/Q	P/P	ON149655
99.	48 AB	Banat's White	F	7		G/G	W/W	T/T	G/G	I/I	H/H	N/N	R/R	R/R	P/P	S/S	R/R	I/I	Q/Q	P/P	ON149656
100.	49 AB	Banat's White	F	1	Yes	G/G	W/W	T/T	G/G	I/I	H/H	N/N	R/R	R/R	P/P	S/S	R/R	I/I	Q/Q	P/P	ON149657

#	Code number	Breed	Sex	Age (years)	Silent mutations	37	102	110	127	142	143	146	151	154	168	173	211	218	222	240	GenBank ID
101.	50 AB	Banat’s White	F	7		G/G	W/W	T/T	G/G	I/I	H/H	N/N	R/R	R/R	P/P	S/S	R/R	I/I	Q/Q	P/P	ON149658
102.	51 AB	Banat’s White	F	2	Yes	G/G	W/W	T/T	G/G	I/I	H/H	N/N	R/R	R/R	P/P	S/S	R/R	I/I	Q/Q	P/P	ON149659
103.	52 AB	Banat’s White	F	4		G/G	W/W	T/T	G/G	I/I	H/H	N/N	R/R	R/R	P/P	S/S	R/R	I/I	Q/Q	P/P	ON149660
104.	1A	French Alpine	M	4		G/G	W/W	T/T	G/G	I/I	H/H	N/N	R/R	R/R	P/P	S/S	R/R	I/I	Q/Q	P/S	ON015424
105.	2A	French Alpine	M	4	Yes	G/G	W/W	P/T	G/G	I/M	H/H	N/N	R/R	R/R	P/P	S/S	R/R	I/I	Q/Q	P/P	ON015425
106.	3A	French Alpine	M	4	Yes	G/G	W/W	T/T	G/G	I/I	H/H	N/N	R/R	R/R	P/P	S/S	R/R	I/I	Q/Q	S/P	ON015426
107.	4A	French Alpine	M	4		G/G	W/W	T/T	G/G	I/I	H/H	N/N	R/R	R/R	P/P	S/S	R/R	I/I	Q/Q	P/P	ON015427
108.	5A	French Alpine	M	4		G/G	W/W	T/T	G/G	I/M	H/H	N/N	R/R	R/R	P/P	S/S	R/R	I/I	Q/Q	P/P	ON015428
109.	1 AF	French Alpine	F	8	Yes	G/G	W/W	T/T	G/G	I/I	H/H	N/N	R/R	R/R	P/P	S/S	R/R	I/I	Q/Q	S/P	ON015429
110.	7 AF	French Alpine	F	2	Yes	G/G	W/W	T/T	G/G	I/I	H/H	N/N	R/R	R/R	P/P	S/S	R/R	I/I	Q/Q	S/P	ON015430
111.	8 AF	French Alpine	F	2		G/G	W/W	T/T	G/G	I/I	H/H	N/N	R/R	R/R	P/P	S/S	R/R	I/I	K/K	S/S	ON015431
112.	9 AF	French Alpine	F	3		G/G	W/W	T/T	G/G	I/I	H/H	N/N	R/R	R/R	P/P	S/S	R/R	I/I	Q/Q	P/P	ON015432
113.	12 AF	French Alpine	F	1	Yes	G/G	W/W	T/T	G/G	I/I	H/H	N/N	R/R	R/R	P/P	S/S	R/R	I/I	Q/Q	S/P	ON015433
114.	14 AF	French Alpine	F	2		G/G	W/W	T/T	G/G	I/I	H/H	N/N	R/R	R/R	P/P	S/S	R/R	I/I	Q/Q	S/S	ON015434
115.	16 AF	French Alpine	F	5	Yes	G/G	W/W	T/T	G/G	I/I	H/H	N/N	R/R	R/R	P/P	S/S	R/R	I/I	Q/Q	S/P	ON015435
116.	17 AF	French Alpine	F	7	Yes	G/G	W/W	T/T	G/G	I/I	H/H	N/N	R/R	R/R	P/P	S/S	R/R	I/I	Q/Q	S/P	ON015436

#	Code number	Breed	Sex	Age (years)	Silent mutations	37	102	110	127	142	143	146	151	154	168	173	211	218	222	240	GenBank ID
117.	21 AF	French Alpine	F	3	Yes	G/G	W/W	T/T	G/G	I/I	H/H	N/N	R/R	R/R	P/P	S/S	R/R	I/I	Q/Q	S/P	ON015437
118.	22 AF	French Alpine	F	3		G/G	W/W	T/T	G/G	I/I	H/H	N/N	R/R	R/R	P/P	S/S	R/R	I/I	K/K	S/S	ON015438
119.	23 AF	French Alpine	F	3		G/G	W/W	T/T	G/G	I/I	H/H	N/N	R/R	R/R	P/P	S/S	R/R	I/I	K/K	S/S	ON015439
120.	24 AF	French Alpine	F	2	Yes	G/G	W/W	T/T	G/G	I/I	H/H	N/N	R/R	R/R	P/P	S/S	R/R	I/I	Q/Q	S/P	ON015440
121.	25 AF	French Alpine	F	2	Yes	G/G	W/W	T/T	G/G	I/I	H/H	N/N	R/R	R/R	P/P	S/S	R/R	I/I	K/K	S/P	ON015442
122.	26 AF	French Alpine	F	3		G/G	W/W	T/T	G/G	I/I	H/H	N/N	R/R	R/R	P/P	S/S	R/R	I/I	Q/Q	S/S	ON015443
123.	27 AF	French Alpine	F	2	Yes	G/G	W/W	T/T	G/G	I/I	H/H	N/N	R/R	R/R	P/P	S/S	R/R	I/I	Q/Q	P/P	ON015444
124.	28 AF	French Alpine	F	1		G/G	W/W	T/T	G/G	I/I	H/H	N/N	R/R	R/R	P/P	S/S	R/R	I/I	Q/Q	S/S	ON015445
125.	29 AF	French Alpine	F	3	Yes	G/G	W/W	T/T	G/G	I/I	H/H	N/N	R/R	R/R	P/P	S/S	R/R	I/I	Q/Q	P/P	ON015446
126.	30 AF	French Alpine	F	3	Yes	G/G	W/W	T/T	G/G	I/I	H/H	N/N	R/R	R/R	P/P	S/S	R/R	I/I	K/K	S/P	ON015447
127.	31 AF	French Alpine	F	2		G/G	W/W	T/T	G/G	I/I	H/H	N/N	R/R	R/R	P/P	S/S	R/R	I/I	Q/Q	P/P	ON084821
128.	32 AF	French Alpine	F	3	Yes	G/G	W/W	T/T	G/G	I/I	H/H	N/N	R/R	R/R	P/P	S/S	R/R	I/I	Q/Q	P/P	ON084822
129.	33 AF	French Alpine	F	2		G/G	W/W	T/T	G/G	I/I	H/H	N/N	R/R	R/R	P/P	S/S	R/R	I/I	K/K	S/S	ON084823
130.	34 AF	French Alpine	F	3		G/G	W/W	T/T	G/G	I/I	H/H	N/N	R/R	R/R	P/P	S/S	R/R	I/I	Q/Q	P/P	ON084824
131.	35 AF	French Alpine	F	4	Yes	G/G	W/W	T/T	G/G	I/I	H/H	N/N	R/R	R/R	P/P	S/S	Q/R	I/I	Q/Q	S/P	ON084825
132.	36 AF	French Alpine	F	7	Yes	G/G	W/W	T/T	G/G	I/I	H/H	N/N	R/R	R/R	P/P	S/S	R/R	I/I	Q/Q	P/P	ON084826

#	Code number	Breed	Sex	Age (years)	Silent mutations	37	102	110	127	142	143	146	151	154	168	173	211	218	222	240	GenBank ID
133.	37 AF	French Alpine	F	4	Yes	G/G	W/W	T/T	G/G	I/I	H/H	N/N	R/R	R/R	P/P	S/S	R/R	I/I	Q/Q	P/P	ON084827
134.	38 AF	French Alpine	F	4		G/G	W/W	T/T	G/G	I/I	H/H	N/N	R/R	R/R	P/P	S/S	R/R	I/I	Q/Q	S/S	ON084828
135.	39 AF	French Alpine	F	4	Yes	G/G	W/W	T/T	G/G	I/I	H/H	N/N	R/R	R/R	P/P	S/S	Q/R	I/I	H/Q	S/P	OM988075
136.	40 AF	French Alpine	F	4	Yes	G/G	W/W	T/T	G/G	I/I	H/H	N/N	R/R	R/R	P/P	S/S	R/R	I/I	Q/Q	P/P	ON084829
137.	41 AF	French Alpine	F	2		G/G	W/W	T/T	G/G	I/I	H/H	N/N	R/R	R/R	P/P	S/S	R/R	I/I	Q/Q	P/P	ON084830
138.	42 AF	French Alpine	F	2	Yes	G/G	W/W	T/T	G/G	I/I	H/H	N/N	R/R	R/R	P/P	S/S	R/R	I/I	Q/Q	P/P	ON084831
139.	43 AF	French Alpine	F	2		G/G	W/W	T/T	G/G	I/I	H/H	N/N	R/R	R/R	P/P	S/S	R/R	I/I	Q/Q	P/P	ON084832
140.	44 AF	French Alpine	F	3		G/G	W/W	T/T	G/G	I/M	H/H	N/N	R/R	R/R	P/P	S/S	R/R	I/I	Q/Q	P/P	ON084833
141.	45 AF	French Alpine	F	2	Yes	G/G	W/W	T/T	G/G	I/I	H/H	N/N	R/R	R/R	P/P	S/S	R/R	I/I	K/K	P/P	ON084834
142.	46 AF	French Alpine	F	2		G/G	W/W	T/T	G/G	I/M	H/H	N/N	R/R	R/R	P/P	S/S	R/R	I/I	Q/Q	P/P	ON084835
143.	47 AF	French Alpine	F	2		G/G	W/W	T/T	G/G	I/I	H/H	N/N	R/R	R/R	P/P	S/S	Q/R	I/I	Q/Q	S/P	ON084836
144.	48 AF	French Alpine	F	4		G/G	W/W	T/T	G/G	I/M	H/H	N/N	R/R	H/R	P/P	S/S	R/R	I/I	Q/Q	S/P	ON084837
145.	49 AF	French Alpine	F	6		G/G	W/W	T/T	G/G	I/I	H/H	N/N	R/R	R/R	P/P	S/S	R/R	I/I	Q/Q	P/P	ON084838
146.	50 AF	French Alpine	F	6		G/G	W/W	T/T	G/G	I/I	H/H	N/N	R/R	R/R	P/P	S/S	R/R	I/I	Q/Q	P/P	ON084839
147.	51 AF	French Alpine	M	1		G/G	W/W	T/T	G/G	I/I	H/H	N/N	R/R	R/R	P/P	S/S	R/R	I/I	Q/Q	P/P	ON084840
148.	52 AF	French Alpine	M	1		G/G	W/W	T/T	G/G	I/I	H/H	N/N	R/R	R/R	P/P	S/S	R/R	I/I	Q/Q	S/S	ON084841

#	Code number	Breed	Sex	Age (years)	Silent mutations	37	102	110	127	142	143	146	151	154	168	173	211	218	222	240	GenBank ID
149.	53 AF	French Alpine	M	1		G/G	W/W	T/T	G/G	I/I	H/H	N/N	R/R	R/R	P/P	S/S	R/R	I/I	Q/Q	P/P	ON084842
150.	54 AF	French Alpine	M	1		G/G	W/W	T/T	G/G	I/I	H/H	N/N	R/R	R/R	P/P	S/S	R/R	I/I	Q/Q	P/P	ON084843
151.	55 AF	French Alpine	M	1		G/G	W/W	T/T	G/G	I/I	H/H	N/N	R/R	R/R	P/P	S/S	R/R	I/I	Q/Q	P/P	ON084844
152.	56 AF	French Alpine	M	1		G/G	W/W	T/T	G/G	I/I	H/H	N/N	R/R	R/R	P/P	S/S	R/R	I/I	Q/Q	P/P	ON084845
153.	57 AF	French Alpine	M	1		G/G	W/W	T/T	G/G	I/I	H/H	N/N	R/R	R/R	P/P	S/S	R/R	I/I	Q/Q	P/S	ON084846
154.	58 AF	French Alpine	M	1	Yes	G/G	W/W	T/T	G/G	I/I	H/H	N/N	R/R	R/R	P/P	S/S	R/R	I/I	K/K	S/P	ON084847
155.	59 AF	French Alpine	M	1	Yes	G/G	W/W	T/T	G/G	I/I	H/H	N/N	R/R	R/R	P/P	S/S	R/R	I/I	Q/Q	P/P	ON084848
156.	60 AF	French Alpine	M	1		G/G	W/W	T/T	G/G	I/I	H/H	N/N	R/R	R/R	P/P	S/S	R/R	I/I	Q/Q	S/S	ON084849
